# Online Health Information for Penile Prosthesis Implants Lacks Quality and Is Unreadable to the Average US Patient

**DOI:** 10.7759/cureus.34240

**Published:** 2023-01-26

**Authors:** Benjamin Plambeck, Jinfeng Jiang, Jesus Perez, Brittany E Wordekemper, David Fu, Alexandra Colvin, Christopher M Deibert

**Affiliations:** 1 Department of Urology, University of Iowa Hospitals and Clinics, Iowa City, USA; 2 Department of Urology, Washington University School of Medicine, St. Louis, USA; 3 Department of Urology, University of Nebraska Medical Center, Omaha, USA

**Keywords:** consumer health information, discern, penile prosthesis, readability, penile implantation, erectile dysfunction

## Abstract

Background: Online health information (OHI) has become widely accessible and affects patient decisions regarding their healthcare. The purpose of this study was to assess the readability, quality, and accuracy of information available to patients online about penile prosthesis implants (PPIs).

Methods: We performed a Google search using the keywords “penile implant” and “penile prosthesis.” The first 30 search results for both terms were analyzed, and advertisements, news articles, duplicates, and videos were excluded. Websites were categorized as institutional, commercial, and personal/patient support. Readability of each website was determined using the Flesch-Kincaid grade level (FKGL) readability formula within the readable tool. Quality was measured by Health On the Net (HON) certification status and the DISCERN scoring method. For website accuracy, a score of 1-4 (1=0-25%, 2=25-50%, 3=50-75%, and 4=75-100%) was assigned.

Results: Forty-four websites met the criteria (23 institutional, 12 commercial, and 9 personal/patient support). The mean total FKGL score was 9.55. No statistical difference was detected between mean FKGL for each website category (p=0.69). Only eight websites (18%) scored ≤8th-grade reading level (average US adult level), while 36 (82%) were >8th-grade level. Mean total DISCERN sum score was 39.74/75, with no statistical difference in mean DISCERN score between website types (p=0.08). Over half (55%) of the websites were defined as “very poor” or “poor” quality by DISCERN scoring. Mean total overall quality rating was 2.67/5. HON certification was verified for only nine websites (20%). Twenty-five percent of websites were classified as 0-25% accurate, 23% were 25-50% accurate, 30% were 50-75% accurate, and 23% were 75-100% accurate.

Conclusion: Most information on the Internet about PPIs is reasonably accurate; however, the majority of websites are deficient in quality and unreadable to the average patient, irrespective of website type.

## Introduction

In today’s technological era, 93% of US adults utilize the Internet and online health information (OHI) has become widely accessible [1]. As a result, patients are more frequently pursuing online answers regarding their health [2,3] with search engines like Google [4]. Additionally, over two-thirds of patients on the Internet reported their treatment decisions were affected by online searches [5]. The availability of OHI allows patients to privately investigate sensitive health topics with ease; however, the quality and accuracy of OHI are not regulated and can lead to negative outcomes for misinformed patients [6,7]. Furthermore, even accurate, high-quality information is useless if unreadable to patients.

The average adult patient in the US reads at or below the 8th-grade level [8,9], and around 33% of adults in the US have low health literacy and struggle to apply health information [10]. Thus, it has been recommended OHI be written at the 6th to 8th-grade level [11,12]. Additionally, many patients have trouble discerning between low- and high-quality health information on the Internet [6]. In light of nearly ubiquitous Internet use among Americans, it is critical that online information available to patients for health-related topics be of high quality.

Erectile dysfunction (ED) affects over 152 million men worldwide [13] and significantly impacts patient quality of life [14]. Understandably, ED was the most popular Google search term related to men’s health for the past five years [15]. Penile prosthesis implant (PPI) surgery is an effective treatment option for ED with satisfactory rates above 90% [14,16]. Although surgery was classically considered third-line therapy for ED [17], the 2018 American Urological Association guidelines for ED now suggest a shared decision-making model where surgical options are considered first-line along with medical therapy [18]. Patients considering PPIs as a treatment for ED should have access to high-quality, accurate, and readable information to support their decision-making.

Previous studies have evaluated the readability, quality, and accuracy of OHI for various other urological diseases and treatments [3,12,19-22]. However, to our knowledge, no study has explored the OHI for PPIs. Thus, the question remains as to whether OHI for PPIs is readable, high-quality, and accurate. The purpose of this study was to assess the readability, quality, and accuracy of health information available to patients online regarding penile prosthesis implants.

## Materials and methods

We utilized the Google search engine to explore the terms “penile implant” and “penile prosthesis” in August 2020, and the first 30 websites were analyzed for both search terms. Paid advertisements, news articles, duplicates, and videos were excluded. Each website was categorized as institutional (university or hospital website), commercial (private business), or personal/patient support.

The readability of each website was assessed using the readable tool, which was accessed through https://readable.com. All educational text was copied from each website into the tool, which used the validated Flesch-Kincaid grade level (FKGL) readability formula to evaluate readability of the entered text. The FKGL formula is a commonly employed readability test and uses mean word and sentence length to produce a score that corresponds with reading grade level [23].

Website quality was determined by two separate individuals using the DISCERN instrument, a validated, 16-question tool made to evaluate the quality of health information regarding treatment choices [24,25]. All 16 questions are subjectively answered with a score of 1-5 and aid in determining whether a source is reliable and of good quality. The last question of this tool constitutes an overall quality rating of the website and is based on the majority of scores from questions 1-15. A score of 1, 3, or 5 on question 16 corresponds with “serious or extensive shortcomings,” “potentially important but not serious shortcomings,” and “minimal shortcomings,” respectively [24]. Our study determined website quality based on the totaled DISCERN scores (max of 75) from questions 1-15 (DISCERN sum score). The two individual scorer’s results were averaged and then analyzed to mitigate subjectivity. Websites were then defined as very poor (15-26), poor (27-38), fair (39-50), good (51-62), or excellent (63-75) quality from the averaged sum scores. This replicates an approach used by multiple studies to evaluate quality with the DISCERN instrument [3,12,22,26]. In addition, Health On the Net (HON) certification, which signifies a website has met specific quality standards for OHI, was verified for websites to further assess quality [27].

The accuracy of each website was also measured. This was determined by two individual urologists, who assigned websites a score of 1-4 (1=0-25% accurate, 2=25-50% accurate, 3=50-75% accurate, 4=75-100% accurate). The results from the two urologists were averaged prior to analysis.

Differences in means between website categories for readability, quality, and accuracy were assessed using one-way analysis of variance (ANOVA) with the Microsoft Excel 2021 Analysis Toolpak (Redmond, WA: Microsoft Corporation). A p-value of <0.05 was considered statistically significant.

## Results

Of the 60 websites accessed from our search, 44 met inclusion criteria and were analyzed. Of these websites, the majority were institutional (23/44, 52%), 12 were commercial (27%), and nine were personal/patient support (20%). HON certification was confirmed for nine websites (20%).

Readability evaluation showed a mean total FKGL score of 9.55, equivalent to above 9th-grade reading level. There was no statistical difference in mean FKGL scores between website categories (p=0.69) (Table 1). Most websites were written at >8th-grade reading level (82%), while 18% scored ≤8th-grade reading level (average US adult reading level). 

**Table 1:** Comparison of readability, quality, and accuracy by website category. N: sample size; RG: range; µ: total mean; P/PS: personal/patient support

Website category (N)	Mean FKGL (RG), µ=9.55	Mean DISCERN sum score (RG), µ=39.74	Mean overall quality rating (RG), µ=2.67	Mean accuracy (RG), µ=2.57
Institutional (23)	9.66 (5.6-14.9)	37.02 (16-69.5)	2.37 (1-5)	2.48 (1-4)
Commercial (12)	9.68 (7.4-12.4)	37.33 (18-57)	2.46 (1-5)	2.42 (1-4)
P/PS (9)	9.07 (5.8-13.0)	49.89 (14-66)	3.72 (1-5)	3.00 (1.5-4)
P-value	0.69	0.08	0.07	0.41

The DISCERN instrument method of assessing quality revealed a mean total DISCERN sum score of 39.74/75, representing “fair” quality. Twenty-three percent of websites were defined as “very poor,” 32% were “poor,” 23% were “fair,” 14% were “good,” and 9% were “excellent.” No statistical difference was detected in mean DISCERN sum scores between website categories (p=0.08) (Table 1). The mean total overall quality rating from question 16 was 2.67/5. 39% of websites were defined as having “serious or extensive shortcomings,” 16% had “potentially important but not serious shortcomings,” and 16% had “minimal shortcomings. There was no significant difference in mean overall quality rating between website types (p=0.07) (Table 1).

Analysis of website accuracy revealed a mean total accuracy score of 2.57/4. Twenty-five percent of websites were classified as 0-25% accurate, 23% were 25-50% accurate, 30% were 50-75% accurate, and 23% were 75-100% accurate. There was no significant difference in mean accuracy scores between website categories (p=0.41) (Table 1).

## Discussion

Today, nearly all adults are dependent on the Internet [1], and 68% of patients using the Internet allow online searches to inform their healthcare decisions [5]. Therefore, it is paramount that patients have access to high-quality OHI that is comprehensible and accurate. Erectile dysfunction, a common urologic issue for men [13], is the highest-trending online search topic concerning men’s health [15], and PPI surgery is an excellent treatment option [18] that can provide patients with higher satisfaction rates than medical therapy [16,28]. As a result, it is important to evaluate whether the online information patients are reading on PPIs is acceptable or not. This study found that the currently available online information for PPIs is inadequate.

Assessment of readability revealed the vast majority (82%) of websites are written above the 8th-grade level and, on average, OHI for PPIs is too complex for the average US adult. This finding is supported by previous studies assessing the readability of OHI for other urologic conditions [3,12,21,22]. One prior study that explored the readability of 52 websites on the topic of benign prostatic hyperplasia (BPH) found that the OHI for BPH is written at the 10th-grade reading level and, similar to our study, unreadable to the average US adult. However, they found that readability was significantly worse for academic/institutional websites compared to commercial websites [21]. On the contrary, our findings of inadequate readability were consistent across different website types. As the average American adult reads at the 8th-grade level, our results could indicate that many men suffering from ED may be unable to fully understand their options, leading to potentially suboptimal decision-making [11,12].

Concerning website quality, OHI for PPI had a mean total DISCERN sum score of 39.74/75, barely falling into the “fair” quality range. Other studies have also found “fair” quality OHI for urology topics, albeit at higher ends of the “fair” range [12,22]. Although we found that average quality was at the lowest end of the “fair” range, it should be noted that over half of the websites (55%) were “poor” or “very poor” quality and only 23% were “good” or “excellent.” This indicates an overall lack of quality for the majority of websites. In addition, we looked at website quality through a different lens using DISCERN question 16, and we found that 39% of websites contained “serious or extensive shortcomings” and only 16% had “minimal shortcomings.” This further supports an online quality deficit. What’s more, it is concerning there was no statistical difference in these quality measures between website categories, as the majority of websites were institutional and would be expected to be reliable sources of OHI. Finally, only nine websites (20%) were HON certified, which has been shown by prior studies to signify “good” quality [12]. Altogether, our findings suggest most online material patients read on PPI is of low quality and an untrustworthy source of information.

With regard to accuracy, two urologists at our institution determined the mean total accuracy score to be 2.57/4. The majority (53%) of websites were deemed 50-100% accurate, with the highest number of websites falling into the 50-75% range. This has been replicated by another urological study and it indicates that the accuracy of online material for PPIs is somewhat reasonable [3].

The findings of our study are important, as the Internet has changed how patients make healthcare decisions and interact with physicians. Today, doctors even find themselves needing to explain OHI to patients to help them understand their search results [29]. Yet, if patients are viewing poor-quality information online, they could still become misled and more likely to make suboptimal decisions. As clinic time with patients is typically short, clinicians should be aware of the OHI in their field and know how to direct patients to reliable sources that are readable and accurate. In addition, producing acceptable online sources for patients to view at their leisure is a great option for physicians to maintain some control over the OHI their patients see. This would further assist in providing high-value care for patients, even after visits conclude. Clinicians could easily accomplish this by applying the exact same readability and quality tools utilized in this study before publishing OHI on the Internet. A potential way readability scores could be improved is by incorporating easy-to-understand illustrations, which may simplify complex material and prevent the use of difficult terminology. With regards to quality, a common downfall for the PPI websites with low DISCERN scores was a lack of cited sources. Thus, to improve quality, websites should always contain references for any material discussed.

This study is not without limitations. First, the readability of all websites could not be fully assessed, as some websites included figures, pictures, or videos that were unable to be analyzed with the Readable tool and may have contributed meaningfully to patient comprehension. Second, although the DISCERN instrument is a valid and widely utilized method of measuring health information quality, it is a subjective rather than objective scoring method [3,12,22,24-26]. To help decrease this subjectivity, two individuals independently scored each website for quality and their results were averaged. Third, the authors acknowledge that the lack of statistical significance between website categories could have been due to the small sample size of the websites analyzed. However, it has been shown that 75% of Internet users do not click past the first page of a Google search [30]. Thus, in order to make our search more applicable to what most patients will view in their searches for OHI, we kept the analysis to the first three pages of our Google search. Finally, although the vast majority of the U.S. population only speaks English at home and Google is by far the most commonly used online search engine, we do acknowledge that our study has a bias toward English-speaking patients who exclusively utilize Google.

## Conclusions

While most online health information regarding penile prosthesis implants is reasonably accurate, the majority of websites are lacking in quality and incomprehensible to the average US adult patient. These findings hold true across website types, and urologists should be aware of this online inadequacy. Furthermore, future work should focus on improving the readability and quality of online information for penile prosthesis implants in order to optimize the ability of patients with erectile dysfunction to make informed healthcare decisions.
